# Hæmorrhagie Anæmia in Cow

**Published:** 1892-05

**Authors:** W. H. Ridge


### HÆMORRHAGIE ANÆMIA IN COW.

By W. H. Ridge, V. M. D.

April 17th I was called to see a cow suffering from mammitis; on arrival I found the animal recumbent, in a natural position, with an udder enormously swollen, which pitted on pressure. She had been in this condition for two days; finding the teat ulcerated badly, I returned home after a teat tube without making further examination; on making my second visit, within one half hour, I found her dead. It is needless to say I was surprised.

I now got the following history: The animal had been ailing for some three months, sometimes missing her meals for a few days, back arched, at times, owner had given some "condition powders," when she would apparently recover, to be soon in the same condition, becoming more and more emaciated. Yet she milked fairly well, and at no time did she show signs of serious sickness until this mammitis, which was only of a few days duration, but very acute.

Autopsy, a few minutes after death. On incision, muscles showed marked anæmia; on cutting the blood vessels, found blood fluid and watery, and in small amount; œdematous spots scattered over body, which, on cutting, let a clear serum escape; serous membrane of abdominal organs normal; rumen moderately filled; reticulum normal; omasum, hard, dry, and one of the centre leaves near the free edge, had attached a papillonia (soft), about 3½ inches long by 2 inches wide, it was a deudritic tumor (microscope verified the diagnosis of papillonia); all the other organs normal, except udder, which, on opening, allowed a large quantity of blood to escape; this was a hæmorrhage around the gland, between abdomen and gland, extending down to near the teat; gland structures greatly inflamed on one side, on opening sinuses a chocolate colored fluid escaped.

It was very evident that the death was caused by the hæmorrhage, owing to the anæmic condition weakening the blood vessels; the anæmic condition was caused by the papillonia causing indigestion.

